# Effect of Angiotensin II and Small GTPase Ras Signaling Pathway Inhibition on Early Renal Changes in a Murine Model of Obstructive Nephropathy

**DOI:** 10.1155/2014/124902

**Published:** 2014-07-03

**Authors:** Ana B. Rodríguez-Peña, Isabel Fuentes-Calvo, Neil G. Docherty, Miguel Arévalo, María T. Grande, Nélida Eleno, Fernando Pérez-Barriocanal, José M. López-Novoa

**Affiliations:** ^1^Unidad de Fisiopatología Renal y Cardiovascular, Instituto “Reina Sofía” de Investigación Nefrológica, Fundación Renal Iñigo Alvarez de Toledo, Departamento de Fisiología y Farmacología, Universidad de Salamanca, Edificio Departamental, Campus Miguel de Unamuno, 37007 Salamanca, Spain; ^2^Centro de Investigación del Cáncer (CSIC), Universidad de Salamanca, 37007 Salamanca, Spain; ^3^Diabetes Complications Research Centre, Conway Institute of Biomolecular and Biomedical Research, University College Dublin, Belfield, Dublin 4, Ireland; ^4^Instituto de Investigaciones Biomédicas de Salamanca (IBSAL), 37007 Salamanca, Spain; ^5^Departamento de Anatomía e Histología Humanas, Universidad de Salamanca, 37007 Salamanca, Spain; ^6^Universidad Francisco de Vitoria, 28223 Madrid, Spain

## Abstract

Tubulointerstitial fibrosis is a major feature of chronic kidney disease. Unilateral ureteral obstruction (UUO) in rodents leads to the development of renal tubulointerstitial fibrosis consistent with histopathological changes observed in advanced chronic kidney disease in humans. The purpose of this study was to assess the effect of inhibiting angiotensin II receptors or Ras activation on early renal fibrotic changes induced by UUO. Animals either received angiotensin II or underwent UUO. UUO animals received either losartan, atorvastatin, and farnesyl transferase inhibitor (FTI) L-744,832, or chaetomellic acid A (ChA). Levels of activated Ras, phospho-ERK1/2, phospho-Akt, fibronectin, and *α*-smooth muscle actin were subsequently quantified in renal tissue by ELISA, Western blot, and/or immunohistochemistry. Our results demonstrate that administration of angiotensin II induces activation of the small GTPase Ras/Erk/Akt signaling system, suggesting an involvement of angiotensin II in the early obstruction-induced activation of renal Ras. Furthermore, upstream inhibition of Ras signalling by blocking either angiotensin AT1 type receptor or by inhibiting Ras prenylation (atorvastatin, FTI o ChA) reduced the activation of the Ras/Erk/Akt signaling system and decreased the early fibrotic response in the obstructed kidney. This study points out that pharmacological inhibition of Ras activation may hold promise as a future strategy in the prevention of renal fibrosis.

## 1. Introduction

Renal interstitial fibrosis is a common histopathological endpoint in all forms of progressive renal diseases independently of their etiology. Unilateral ureteral obstruction (UUO) is a well-established experimental model in mice leading to tubulointerstitial fibrosis in the ligated kidney [1, 2]. Increased synthesis of angiotensin II (Ang II) has been implicated as playing a causative role in progression of kidney damage in obstructive nephropathy [3–5]. Ang II is directly profibrotic but also acts as a proinflammatory cytokine in the kidney through the activation of the nuclear factor-*κ*B family of transcription factors, which in turn, induces an autocrine response enhancing both Ang II and tumor necrosis factor alpha production [6]. By promoting tubulointerstitial infiltration of inflammatory cells, Ang II also contributes to the onset and progression of renal damage in UUO [6]. However, studies examining the effect of directly targeting Ang II in UUO-induced renal damage have yielded contradictory results. Thus, whereas it has been reported that Ang II receptor blockade or angiotensin-converting enzyme inhibition abrogates fibrosis deposition and myofibroblast proliferation in some studies of obstructive nephropathy [7–9], in other studies these interventions have been shown to aggravate renal damage after UUO [10–12]. These differences may be due to whether obstruction was partial or complete but they also suggest that optimal strategies may be better targeted downstream of the Ang II receptor complex, focused on those pathways that are implicated in the proinflammatory and profibrotic responses to Ang II.

Actions of Ang II are mediated via the Ang II type I (AT1) and type II G-protein coupled receptors (GPCR), as well as the receptor tyrosine kinases (RTK)s [13, 14]. Specific profibrotic actions of Ang II are implicated in the appearance of alpha-smooth muscle actin (*α*-SMA)-positive fibroblasts or myofibroblasts and fibronectin accumulation which are considered prominent features of fibrosis development in the obstructed kidney [15, 16].

Ras monomeric GTPases play a major role in the control of proliferation, differentiation, and cell death, connecting activated RTKs and GPCRs to the effector pathways Raf/mitogen-activated protein kinase (MAPK)/extracellular signal regulated kinases 1 and 2 (ERK1/2) and phosphatidylinositol-3 kinase (PI3 K)-Akt [17]. Many growth factors are known to activate intracellular signaling pathways that converge on Ras activation, including Ang II which has been reported to stimulate renal Ras/MAPK pathway* in vivo* and* in vitro* [18, 19]. Thus, Muthalif et al. [18] have demonstrated that Ang II infusion for 6 days induces hypertension and renal Ras activation, and both phenomena are reversed by administration of a farnesyl transferase inhibitor (FTI). However, the effects of acute Ang II administration on Ras activation have not been assessed.

Activation of Ras and its effectors ERK1/2 and PI3 K/Akt has been reported as mediators in progressive renal damage [20, 21]. Activation of Ras signaling pathway occurs after early UUO [22, 23], demonstrating a contribution of Ras downstream effectors to renal injury with a main involvement of ERK1/2 in apoptotic events and Akt in proliferative and fibrotic response [23]. There are several Ras isoforms (H-, N- and K-Ras) with different functional properties in fibrotic processes and in fibroblast biology [24–26]. Thus, we have observed that H-Ras knock-out (KO) mice show lower fibrosis after UUO [27], whereas in embryonic fibroblasts obtained from H-Ras or N-Ras KO mice, fibronectin and collagen synthesis were higher and proliferation and migration were lower than in wild type fibroblasts [24, 25]. Moreover, K-Ras knock-down decreases stimulated proliferation in renal fibroblasts [28] and inhibits fibrosis in a rat experimental model [29]. It is known that activation of Ras requires several posttranslational modifications that include prenylation, the addition of either the 15-carbon isoprenoid farnesy1 or the 20-carbon isoprenoid geranylgeranyl to cysteine residue(s) at or near the C-termini of Ras proteins, allowing their anchorage to the cell membrane and subsequent activation [30]. Some evidence exists to demonstrate that inhibition of prenylation reduces extracellular matrix production by fibroblasts “*in vitro*” [31]. Thus, we aimed to interrogate the effects of decreasing prenylation on early UUO-induced Ras pathway activation and associated fibrotic responses in the mouse kidney. We used multiple strategies to address the question: (1) by inhibiting the synthesis of farnesyl groups using atorvastatin, an inhibitor of the enzyme 3-hydroxy-3-methylglutaryl coenzyme A (HMG-CoA) reductase-involved in the synthesis of isoprenoid groups required for Ras prenylation [32, 33], (2) by directly inhibiting Ras farnesylation using a farnesyl transferase inhibitor [34], and (3) by treating with chaetomellic acid A, which selectively blocks H-Ras farnesylation [35] with reduced off-target toxicity relative to FTIs [36].

## 2. Methods

### 2.1. Animals and Disease Model

C57BL/6J male mice were kept in a germ-free facility, under controlled environmental conditions (Unidad de Experimentación Animal, University of Salamanca, Spain). Mice were reared on standard chow (Panlab, Barcelona, Spain) and provided with water* ad libitum*. Surgical techniques to produce UUO were performed as previously described on animals at 2 months of age [23]. All procedures were approved by the Committee for Animal Care and Use of the University of Salamanca and complied with the Guide for the Care and Use of Laboratory Animals of National Research Council.

### 2.2. Pharmacological Groups and Drug Administration

*Angiotensin (Ang II) Group.* A group of mice was treated with a single intraperitoneal dose of Ang II (0.8 mg/kg; Sigma, Saint Louis, MO, USA; *n* = 3 per time point) or saline vehicle (NaCl 0.9%; *n* = 3). No surgery was performed in this group of animals and kidneys were removed either 30 minutes, 4, or 12 hours after Ang II administration.

*Losartan Group.* A group of animals received a daily intraperitoneal injection of losartan (40 mg/kg; Du Pont, Wilmington, DE, USA; *n* = 5), whereas the corresponding control group received the vehicle isotonic saline (NaCl 0.9%; *n* = 3), for 4 days. UUO was carried out on the second day of treatment.

*Atorvastatin Group.* A group of mice were treated with atorvastatin calcium (70 mg/kg/day; Pfizer, Madrid, Spain; *n* = 4) by oral gavage, and the corresponding control mice group were treated with carboxymethylcellulose vehicle (Sigma, Saint Louis, MO, USA; *n* = 3), once daily for 6 days. UUO was performed at the fourth day after initiating the treatment.

*Farnesyl Transferase Inhibitor (FTI) Group.* A group of mice received a subcutaneous injection of L-744,832 (40 mg/kg; Biomol Inc, Plymouth Meeting, PA, USA; *n* = 5) while a control group received the vehicle solution (17 mM sodium citrate, 94 mM sodium chloride; pH 5.4; *n* = 3), daily for 6 days. UUO was performed on the fourth day of treatment.

*Chaetomellic Acid A Group.* A group of mice received subcutaneously injected chaetomellic acid A (3 mg/kg/day; Santa Cruz Biotechnology, CA, USA; *n* = 4) during 6 days, whereas a control group received the vehicle solution (17 mM sodium citrate, 94 mM sodium chloride; pH 5.4; *n* = 4). UUO was performed on the fourth day of treatment.

### 2.3. Preparation of Kidney Tissue and Protein Analysis

At endpoint in each group, kidneys were removed under terminal anaesthesia. Methods used for protein analysis, including affinity precipitation of Ras-GTP or ELISA Ras activation Kit (Upstate Biotechnology, MA, USA) and immunodetection of proteins by Western blot and immunohistochemistry, have been already described [23, 25, 27]. As we have previously reported, the amount of loading controls for WB such as tubulin or GAPDH change after UUO [23], and thus we have decided to control strictly the amount of protein loaded instead to perform WB for these proteins.

### 2.4. Statistical Analysis

One-way analysis of variance (ANOVA) was applied for statistical analysis (NCSS 2000 program, Utah, USA). Bonferroni's or Kruskal-Wallis multiple-comparison tests were, respectively, employed for analysis of data with or without normal distribution. Data were expressed as mean ± Standard Error of the Mean (SEM). *P* < 0.05 or *Z* > 1.96 were considered statistically significant.

## 3. Results 

### 3.1. Renal Activation of Ras Signaling Pathway after Short-Term Ang II Infusion

**Figure 1:**
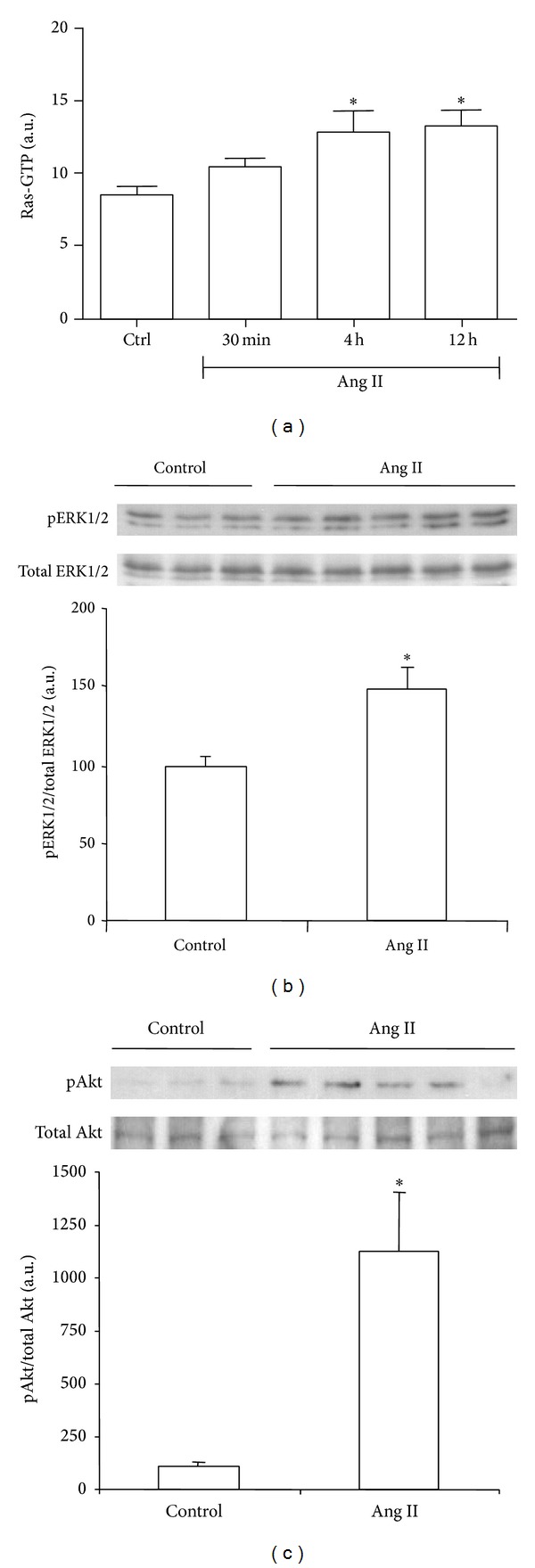
Effect of systemically administrated angiotensin II (Ang II) on Ras signaling pathway. Ras activation was evaluated as Ras GTP by ELISA (a). Phosphorylated (p)-ERK1/2 and p-Akt protein expression were evaluated as the ratio p/total protein by Western blot ((b) and (c), resp.). Bars represent the mean ± SEM of the optical density measured in kidney samples of control saline group (Ctrl; *n* = 3) and angiotensin II-treated animals (Ang II, 0.8 mg/kg; *n* = 3–5/per time point). *P < 0.05 versus control group.

Renal Ras activation, measured by ELISA, was higher both at 4 and 12 hours after single dose Ang II administration than in saline-treated control group (Ctrl; Figure 1).

Western blot analysis also detected an Ang II-induced increase in renal activation of Ras signaling effectors, ERK1/2 and Akt, as demonstrated by measuring the ratio phosphorylated (p)/total protein at 4 hours after Ang II administration (Figures 1(b) and 1(c), resp.).

### 3.2. Effect of AT1 Receptor Antagonist on Ras Activation and Renal Changes after UUO

**Figure 2:**
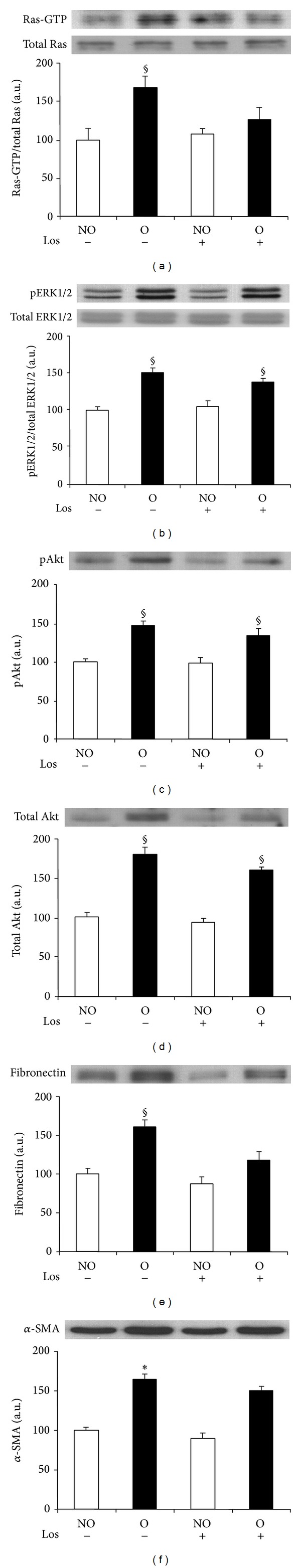
Effect of losartan administration on UUO-induced Ras pathway activation and fibrotic changes analyzed by Western blot. Protein expression of Ras (a), ERK1/2 (b), Akt ((c) and (d)), fibronectin (e), and *α*-SMA (f) detected by immunoblotting. Activation of Ras and ERK1/2 were measured as the ratio phosphorylated (p)/total proteins. Bars represent the mean ± SEM of the optical density measured in nonobstructed (NO) and obstructed (O) kidney samples of saline (*n* = 3) and losartan- (Los 40 mg/kg; *n* = 5) treated animals. ^§^ *P* < 0.05 and *Z > 2.6383 versus NO vehicle-treated kidneys of UUO mice.

We aimed to determine the effect of AT1 receptor antagonist losartan on the activation of Ras/ERK/Akt signaling pathway and the expression of fibronectin and *α*-SMA in kidneys submitted to 3 days of UUO. Western blot analyses showed that levels of Ras-GTP, pERK, pAkt, total Akt, fibronectin, and *α*-SMA were significantly higher in obstructed (O) kidneys than in nonobstructed (NO) kidneys of vehicle-treated animals (Figures 2(a)–2(f)). The increase observed in total Akt levels in kidneys submitted to ureteral obstruction (Figure 2(d)) has been previously described [23]. After receiving losartan treatment, no significant differences were found for levels of activated RAS, fibronectin, and *α*-SMA in O kidneys compared with NO kidneys (Figures 2(a), 2(e) and 2(f), resp.), and a similar pattern was observed for pERK1/2 and pAkt (Figures 2(b) and 2(c), resp.). No differences between NO groups of treated and untreated groups were observed for any of the analyzed proteins.

### 3.3. Effect of Ras Activation Inhibitors on ERK1/2 and Akt Activation and Fibrotic Markers after UUO

Our next objective was to assess whether inhibition of Ras activation after UUO could modulate activation of its downstream effectors, ERK1/2, and Akt, as well as expression of fibronectin and *α*-SMA in ligated kidneys. For this purpose we treated the animals with either the HMG-CoA reductase inhibitor atorvastatin or the farnesyl transferase inhibitor (FTI) L-744,832 or chaetomellic acid A.

**Figure 3:**
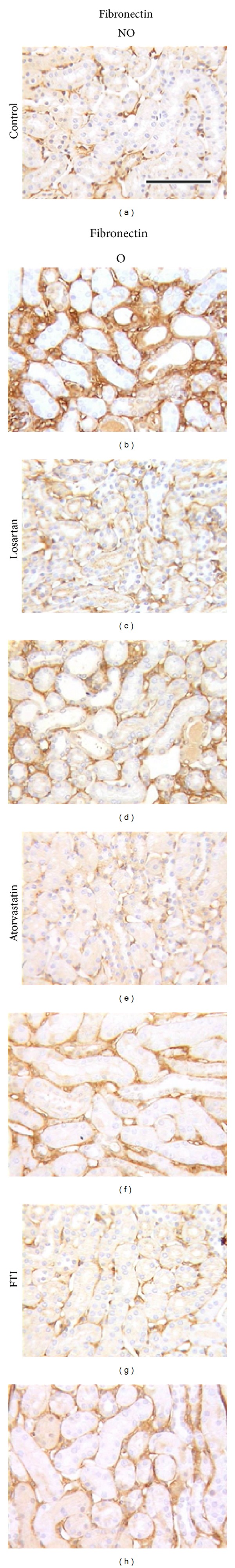
Effect of losartan, atorvastatin, or farnesyl transferase inhibitor (FTI) administration on renal fibronectin expression detected by immunohistochemistry in UUO mice. Representative interstitial sections from nonobstructed (NO) and obstructed (O) kidneys of UUO untreated control mice ((a) and (b)) and UUO mice treated with losartan ((c) and (d)), atorvastatin ((e) and (f)), or FTI ((g) and (h)). Black bar indicates 100 microns in all panels.

**Figure 4:**
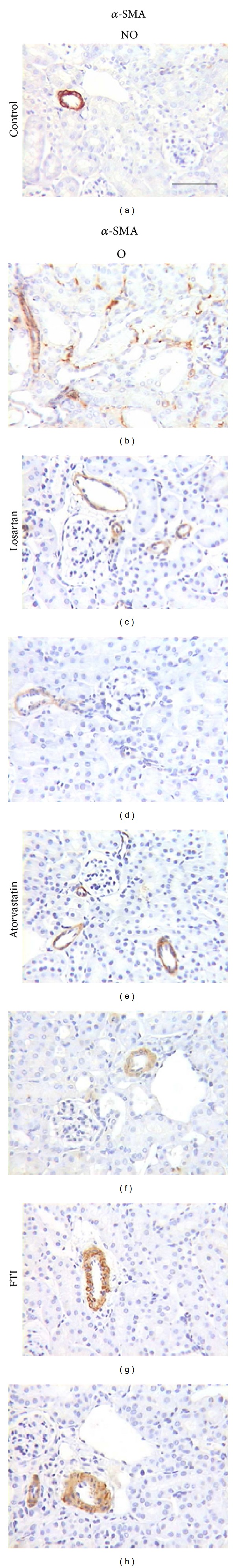
Effect of losartan, atorvastatin, or farnesyl transferase inhibitor (FTI) administration on renal alpha-smooth muscle actin (*α*-SMA) expression detected by immunohistochemistry in UUO mice. Representative interstitial sections from nonobstructed (NO) and obstructed (O) kidneys of UUO untreated control mice ((a) and (b)) and UUO mice treated with losartan ((c) and (d)), atorvastatin ((e) and (f)) or FTI ((g) and (h)). Black bar indicates 100 microns in all panels.

**Figure 5:**
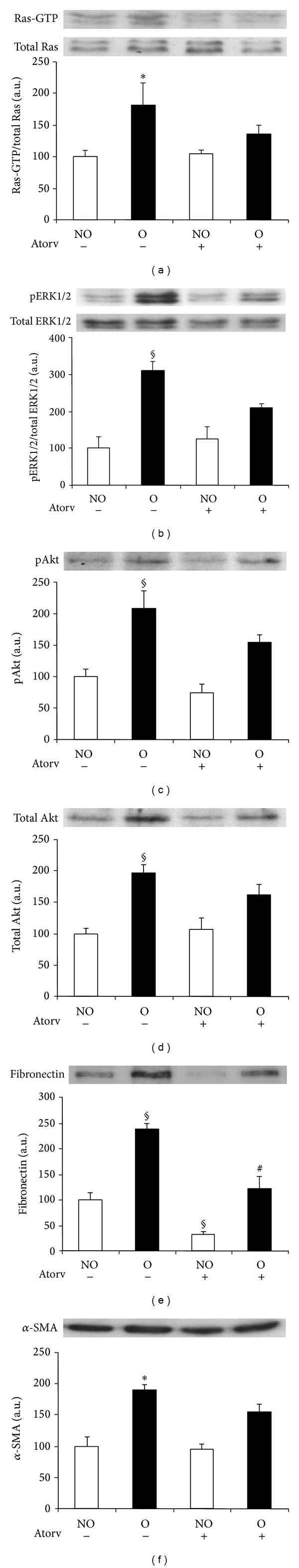
Effect of atorvastatin administration on UUO-induced Ras pathway activation and fibrotic changes analyzed by Western blot. Protein expression of Ras (a), ERK1/2 (b), Akt ((c) and (d)), fibronectin (e), and alpha-smooth muscle actin (*α*-SMA) (f) was detected by immunoblotting. Activation of Ras and ERK1/2 was measured as the ratio phosphorylated/total proteins. Bars represent the mean ± SEM of the optical density measured in nonobstructed (NO) and obstructed (O) kidney samples of vehicle (*n* = 3) and atorvastatin-treated animals (Atorv, 70 mg/kg; *n* = 4). ^§^ *P* < 0.05 and *Z > 1.9600 versus NO vehicle-treated kidneys of UUO mice. ^#^ *P* < 0.05 versus O vehicle-treated kidneys.

Western blot analysis revealed that whereas O kidneys of vehicle-treated mice showed higher levels of Ras-GTP, pERK1/2, pAkt, and total Akt proteins than NO kidneys, the differences between O and NO kidneys for Ras-GTP, pERK1/2, pAkt, and total Akt abundance were not significant (Figures 5(a)–5(d)) in atorvastatin-treated animals (Figures 5(a)–5(d)). Similar results were obtained for fibronectin and *α*-SMA, with lower amounts for both proteins detected in the O kidneys of animals treated with atorvastatin than in O kidneys of vehicle-treated mice (Figures 5(e) and 5(f), resp.). In agreement with these results, immunohistochemistry studies revealed reduced amount of fibronectin (Figures 3(a), 3(b), 3(e) and 3(f)) and interstitial *α*-SMA (Figures 4(a), 4(b), 4(e) and 4(f)) in the O kidneys of atorvastatin-treated mice than in the O kidneys of vehicle-treated animals.

**Figure 6:**
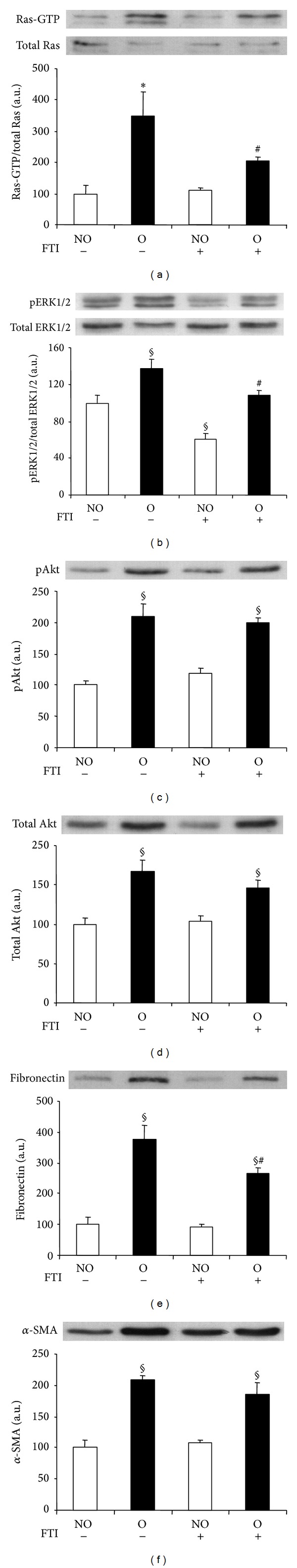
Effect of farnesyl transferase inhibitor (FTI) administration on UUO-induced Ras pathway activation and fibrotic changes analyzed by Western blot. Protein expression of Ras (a), ERK1/2 (b), Akt ((c) and (d)), fibronectin (e), and alpha-smooth muscle actin (*α*-SMA) (f) was detected by immunoblotting. Activation of Ras and ERK1/2 was measured as the ratio phosphorylated/total proteins. Bars represent the mean ± SEM of the optical density measured in nonobstructed (NO) and obstructed (O) kidney samples of vehicle (*n* = 3) and FTI-treated animals (40 mg/kg; *n* = 5). ^§^ *P* < 0.05 and *Z > 2.6383 versus NO vehicle-treated kidneys of UUO mice. ^#^ *P* < 0.05 versus O vehicle-treated kidneys.

Pull-down and Western blot analysis showed that O kidneys of FTI-treated mice presented significantly lower Ras activation than kidneys of vehicle-treated animals (Figure 6(a)). Administration of FTI significantly blunted the obstruction-induced increase in pERK1/2 when compared to control vehicle group, with lower levels of activated protein also found in NO kidneys of FTI (Figure 6(b)). A slight, nonsignificant decrease in the expression of pAkt and total Akt was observed in O kidneys from FTI-treated animals (Figures 6(c) and 6(d), resp.). We observed a marked and significant reduction in fibronectin levels of O kidneys after FTI treatment when compared with kidneys of animals receiving vehicle administration (Figure 6(e)). In agreement with these results, immunohistochemical analysis revealed lower fibronectin staining in the renal cortical interstitium of O kidneys from FTI-treated mice compared with mice receiving the vehicle (Figures 3(a), 3(b), 3(g) and 3(h)). Compared with untreated mice, O kidneys of FTI group showed a mild although nonsignificant reduction in *α*-SMA levels (Figure 6(f)), which correlates with the interstitial amount detected by Figures 4(a), 4(b), 4(g) and 4(h).

**Figure 7:**
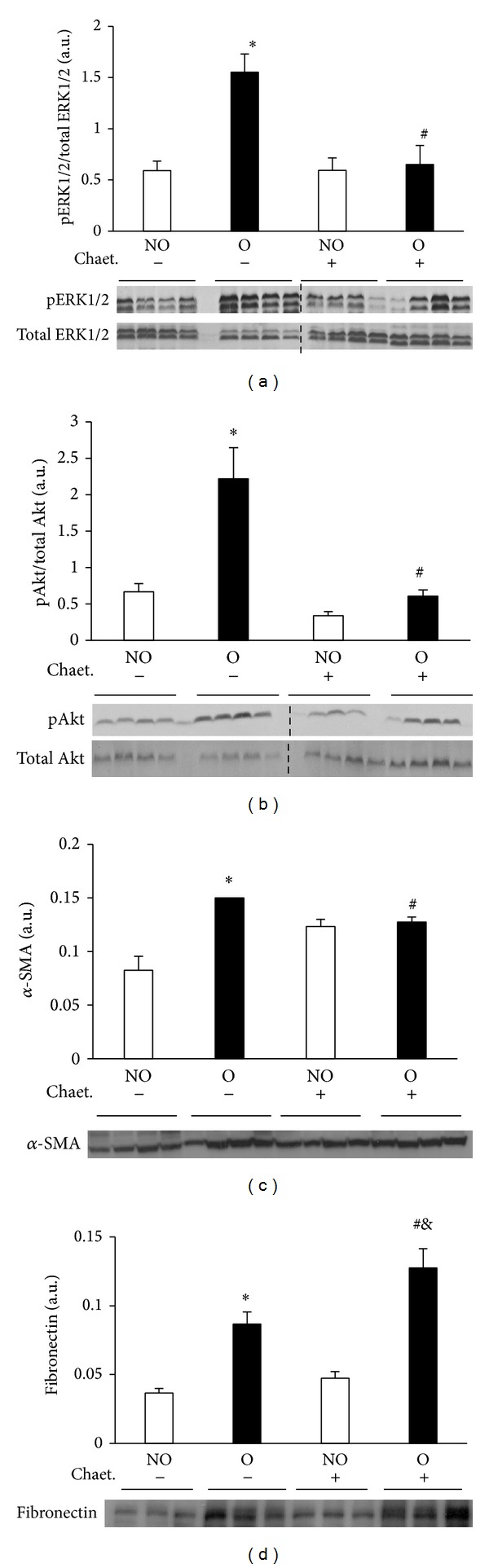
Effect of chaetomellic acid A administration on UUO-induced Ras pathway activation and fibrotic changes analyzed by Western blot. Protein expression of pERK1/2 and ERK1/2 (a), pAkt and Akt (b), alpha-smooth muscle actin (*α*-SMA) (c), and fibronectin (d) was detected by immunoblotting. Bars represent the mean ± SEM of the optical density measured in nonobstructed (NO) and obstructed (O) kidney samples of vehicle (*n* = 4) and chaetomellic acid A-treated animals (Chaet.; 1.5 mg/kg; *n* = 4). *P < 0.01 versus NO vehicle-treated kidneys of UUO mice. ^#^ *P* < 0.01 versus O vehicle-treated kidneys. ^&^ *P* < 0.01 versus NO Chaet-treated kidney.

**Figure 8:**
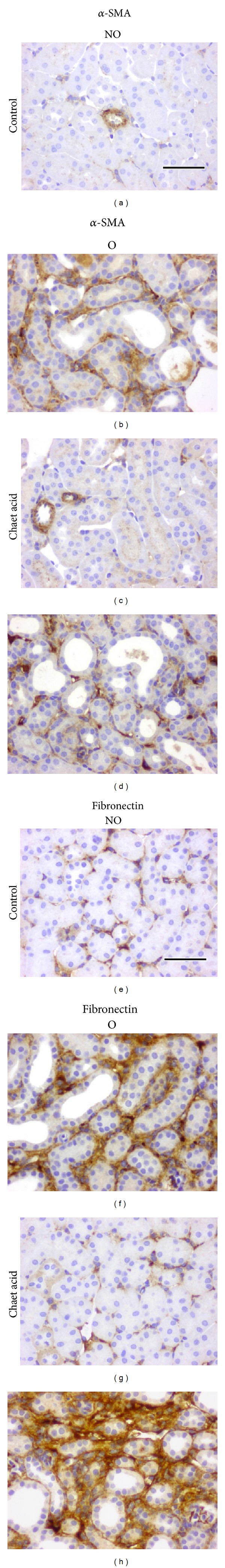
Effect of chaetomellic acid A administration on renal alpha-smooth muscle actin (*α*-SMA) ((a)–(d)) and fibronectin ((e)–(h)) expression detected by immunohistochemistry in UUO mice. Representative cortical interstitial sections from nonobstructed (NO) and obstructed (O) kidneys of UUO mice treated with vehicle (Control) or chaetomellic acid A (Chaet Acid). Black bar indicates 100 microns in all panels.

Western blot analysis showed significantly lower pERK1/2 and pAkt levels in O kidneys from mice that received chaetomellic acid A with respect to O kidneys of control mice (Figures 7(a) and 7(b)). As measured by Western blotting, the expression of *α*-SMA, but not that of fibronectin was significantly lower in O kidneys of mice treated with chaetomellic acid compared with O kidneys of mice treated with vehicle (Figures 7(c) and 7(d), resp.). Immunohistochemistry analysis revealed that in the animals receiving chaetomellic acid, O kidneys showed lower staining for *α*-SMA but not for fibronectin than O kidneys from animals receiving the vehicle alone (Figure 8).

## 4. Discussion

Increased levels of Ang II have been suggested to play a major role in the progression of renal disease induced by experimental UUO [3]. Previous studies have demonstrated that both mRNA and protein levels are increased for renin, angiotensin converting enzyme activity, and Ang II content in the obstructed kidney 1 day after UUO [4]. Additionally,* in vivo* studies have shown renal Ras activation induced by infusion of Ang II for 6 days [18].

Having previously demonstrated that Ras signaling pathway is activated in obstructed kidneys after 3 days of UUO [22, 23, 37], we now demonstrate that systemic Ang II administration in normal mice results in a marked increase of the renal Ras signaling pathway as early as 30 minutes, with larger increases observed 4 hours after administration. Thus, our data demonstrates a rapid activation of the Ras pathway induced by UUO or Ang II administration. Furthermore, by blocking the AT1 receptor by losartan administration, levels of activated Ras in obstructed kidneys were markedly reduced. Taken together, these results suggest that Ang II leads to AT1 receptor dependent stimulation of Ras in early renal injury post-UUO.

Fibronectin is a major component of the pathological extracellular matrix (ECM) in tubulointerstitial fibrosis that serves as a fibroblast chemoattractant and scaffold protein for the deposition of other ECM proteins [38]. Furthermore, the fibronectin scaffold has been demonstrated to be involved in the differentiation of fibroblasts to the *α*-SMA positive myofibroblasts [39], a key step in UUO-induced renal fibrosis [16]. Since inhibition of UUO-induced Ras activation by losartan treatment attenuates increases in fibronectin and *α*-SMA expression in the obstructed kidney, a potential role for Ras beyond mere association could be suggested in relation to the early stages of Ang II-mediated renal fibrosis.

Considering all this data, it could be suggested that in the early stages of UUO the profibrotic effects of AT1 receptor-mediated Ang II are regulated, at least in part, via a Ras-dependent pathway.

Both downstream effectors of Ras, ERK1/2, and Akt have been implicated in renal damage response in obstructive nephropathy. We have previously reported that activation of Ras and ERK1/2 is significantly higher in the obstructed (O) kidneys than in nonobstructed (NO) kidneys 3 days after UUO. The same pattern was observed in UUO-induced expression of p-Akt and total Akt, as demonstrated by Western and Northern blot analysis [22, 23]. Activation of ERK1/2 has been related to interstitial apoptosis and proliferation of tubular cells [23, 40, 41], whereas a role for activated Akt has been reported in early tubulointerstitial cell proliferation and profibrotic events in UUO [23]. Results presented in this study have also shown that inhibition of Ras activation by blocking either the farnesyl transferase or HMG-CoA reductase enzymes is able to reduce UUO-induced MAPK-ERK1/2 signaling pathway in the obstructed kidney. Additionally, administration of atorvastatin or chaetomellic acid A also induced a marked downregulation of PI3 K/Akt pathway. Thus, blockade of Ras activation can provide a new strategy to reduce the renal damage events elicited by activation of ERK1/2 and Akt signaling pathways.

Moreover, our data demonstrate that inhibition of Ras activation by atorvastatin or FTI administration reduces UUO-induced accumulation of fibronectin in the mouse kidney. Atorvastatin data are in agreement with previous studies reporting protective effect of statins in UUO-induced renal fibrosis [42–46].

In addition, atorvastatin, and chaetomellic acid A reduced the increased amount of the marker for myofibroblasts, *α*-SMA, after UUO. At least a part of myofibroblast present in the kidney after UUO may derive from epithelial cells by a process called epithelial-mesenchymal transition (EMT) [16] and there are enough evidences that Ras activation participates in EMT [47, 48]. These results suggest that Ras participates in the initiating molecular events are involved in renal interstitial fibrogenesis induced by ureteral obstruction in mice. We have already reported that H-Ras isoform is able to modulate renal fibrosis and myofibroblast activation following ureteral obstruction in mice [24, 27]. Notably Ras activation has been shown to be involved in EMT of tubular cells to myofibroblasts [49], and specifically H-Ras is involved in TGF-*β*1-induced EMT [50]. In the kidney, chaetomellic acid A selectively inhibits the membrane-bound of H-Ras without affecting the membrane-bound of Ki-Ras or other prenylated intracellular proteins like Rab [51]. Since H-Ras isoform only can be farnesylated, we have used chaetomellic acid A specifically to inhibit the activation of H-Ras isoform. Our results have demonstrated that chaetomellic acid A administration decreased Ras downstream signaling pathway as well as *α*-SMA accumulation in UUO kidneys. Previous studies of our group have shown that H-Ras KO mice show reduced numbers of myofibroblasts in the kidney following UUO [27]. It has been reported that in acute renal ischemia-reperfusion injury in rats the inhibition of the Ras pathway by chaetomellic acid A resulted in a beneficial effect, preserving both renal function and structure [51]. Furthermore, in an experimental murine model of ischemic stroke, chaetomellic acid A administration increased the intracellular concentration of inactive H-Ras, leading to a marked decrease of both superoxide anion production and volume of cerebral necrotic tissue, with the subsequent improved survival of hypoxic neuronal cells [35]. Thus, inhibition of H-Ras isoform specifically by chaetomellic acid A could be used as a potential therapeutic strategy to reduce fibrosis development.

## 5. Conclusions

In summary, our results suggest that increased Ang II production in the obstructed kidney could play a role in Ras/ERK/Akt pathway activation, which in turn, can be involved in early renal fibrosis induced by UUO. Our data also offer evidence of the pharmacological potential of Ras pathway inhibition in preventing the progression of renal tubulo-interstitial fibrosis. Chaetomellic acid A is of particular interest in this regard in relation to the arrest or reversal of renal fibrosis as it acts more specifically than statins and is less toxic than synthetic FTIs.
